# Transformational Leadership and Nursing Retention: An Integrative Review

**DOI:** 10.1155/2024/3179141

**Published:** 2024-07-20

**Authors:** Becky Goens, Natalie Giannotti

**Affiliations:** Faculty of Nursing University of Windsor, Windsor, Canada

## Abstract

**Aim:**

To establish current evidence on the relationship between transformational nursing leadership and turnover intention.

**Background:**

The persistent nursing shortage in healthcare has led to heightened demands for addressing both current needs and the healthcare requirements of a growing population. Recognizing the pivotal role of nursing leadership in fostering retention, this review highlights the influence of positive leadership on nursing staff. *Evaluation*. An integrative review, guided by Whittemore and Knafl's (2005) framework, was conducted using articles sourced from four online databases deducing to an inclusion of sixteen quantitative articles, one systematic review, and one integrative review published between 1992 and 2022. *Key Issues*. The study reveals conflicting evidence regarding the sole impact of transformational leadership on the nursing staff's intention to remain. However, it highlights transformational leadership's ability to enhance job satisfaction and organizational commitment contributes significantly to retention.

**Conclusion:**

Using transformational leadership can effectively bolster nursing staff retention along with promoting other favorable workplace outcomes. *Implications for Nursing Management*. This review underscores the importance of enhancing leadership skills within nursing management. This involves not only fostering transformational leadership but also cultivating positive work-related outcomes to optimize nursing staff retention.

## 1. Introduction

The global nursing shortage has become a prominent conversation in nursing and therefore nursing leadership needs to be diligent in identifying and advocating for change [1, 2]. Understanding leadership's impact on patient satisfaction and care is essential for achieving healthcare objectives [3]. Patient safety outcomes rely on a positive safety culture cultivated by effective leadership to dismantle barriers to care [4]. Various leadership styles, such as transformational, transactional, autocratic, and laissez-faire, have been studied. Nursing leadership significantly influences retention by fostering positive leadership, healthy work environments, job satisfaction, and reducing negative work experiences [5]. Nurse managers empower nurses through positive environments. Certain leadership styles, supported in the literature, promote work satisfaction and turnover intention mitigating absenteeism and increased psychological distress [6].

## 2. Review of the Literature

Nurse retention is a complex issue influenced by various factors, including leadership styles, which can be situationally or organizationally rooted based on specific nursing and healthcare demands [7]. Leadership styles hold significant sway over a nurse's inclination to stay within an organization. Transformational leadership stands out as extensively researched across other disciplines and is considered the best-chosen style for healthcare leadership to encourage staff to provide proficient services with improved morale [8], thus, also producing positive outcomes for organizations, staff, and patients [9]. Five measurable components characterize transformational leadership: behavioral idealized influence, attributed idealized influence, inspirational motivation, intellectual stimulation, and individualized consideration [10]. Attributed idealized influence refers to leaders who display confidence in ways that build respect. Behavioral idealized influence involves discussing values and beliefs, specifying a sense of purpose and emphasizing the importance of a mission. Avolio and Bass [10] also suggest that inspirational motivation uses an energizing attitude for future optimism in achieving goals. Intellectual stimulation pertains to leaders who promote opportunities for professional growth [11]. Finally, individual consideration focuses on the personal needs of each individual, especially those that are seemingly neglected [12]. Such leaders empower and motivate professional growth directly through the provision of a positive environment [6].

Transformational leadership has been linked to improved patient safety, care satisfaction, and decreased adverse events such as medication errors [13, 14]. Crucially, it can mitigate nursing turnover by promoting intentions to stay at work [15]. This “anticipated turnover” strongly correlates with actual turnover, serving as a reliable indicator of future attrition [16]. Retaining experienced nursing staff familiar with unit operations positively impacts patient care outcomes [17], as their departure depletes valuable expertise, skills, and knowledge [7].

## 3. Methods

### 3.1. Design

Guided by Whittemore and Knafl's [1] integrative review framework, this literature review followed their five-stage process: (1) problem identification; (2) comprehensive literature search; (3) evaluation of the data; (4) data analyses; and (5) presentation of synthesized data. The research question was developed using the PICO format which stands for patient, intervention, comparator (if relevant), and outcome [18]. The research question created was as follows: what is the relationship between transformational nursing leadership and turnover intention among staff nurses?

### 3.2. Inclusion and Exclusion Criteria

**Figure 1:**
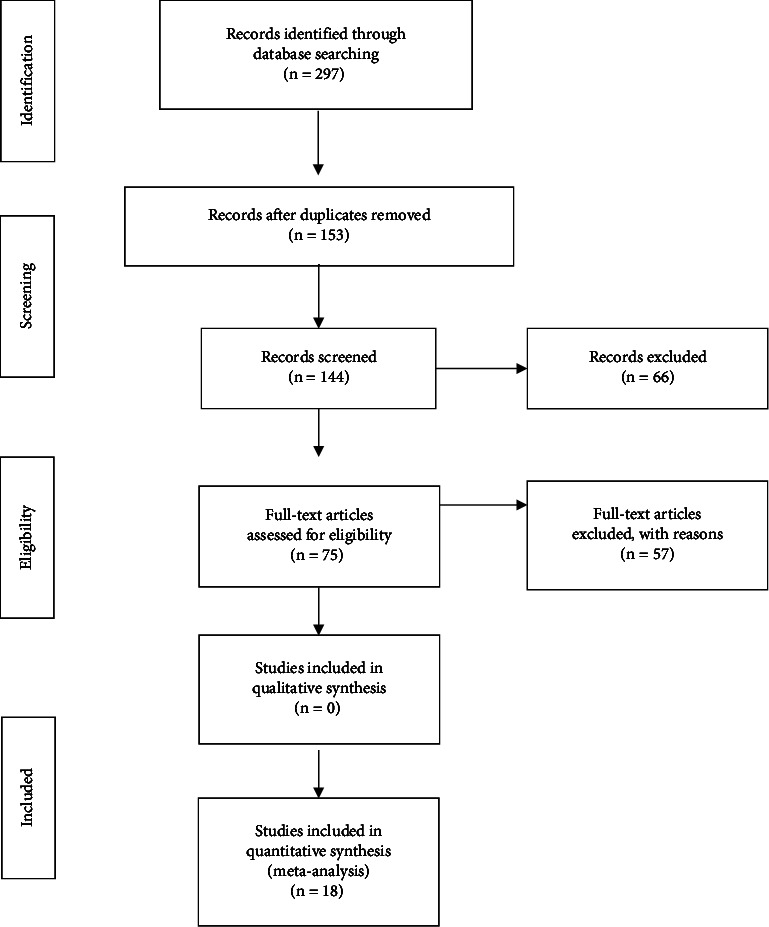
PRISMA flowchart.

Inclusion criteria are as follows: (1) articles must be written in English/translated to English; (2) must be peer-reviewed; and (3) study types must include qualitative, quantitative, mixed-methods, and systematic/integrative/scoping/literature reviews. Exclusion criteria are as follows: (1) opinion pieces, dissertations, thesis, grey literature, letters, and editorials. The Preferred Reporting Items for Systematic Reviews and Meta-Analyses (PRISMA) flow diagram [19] search results are displayed in Figure 1.

### 3.3. Search Strategy

The literature search was carried out in June 2022, using four online databases including Cumulative Index to Nursing and Allied Health Literature (CINAHL), MEDLINE via Ovid, ProQuest Nursing and Allied Health, and PubMed. Search terms were a combination of keywords reported in Table 1.

### 3.4. Search Outcomes and Extraction of Data

Following the literature search, all citations were collected, uploaded, and stored in Covidence, a web-based platform used for conducting comprehensive literature reviews. Covidence is a software that helps streamline the reviewing process, facilitating the screening of citations, reviewing full-text articles, assessing the risk of bias, extracting study characteristics, and creating reports to export [20]. Duplicates were removed using Covidence, followed by a manual review to ensure accuracy. All titles and abstracts were reviewed in conjunction with our inclusion criteria. When an abstract was not present, the study was read in its entirety to determine appropriateness. Full texts of studies were subsequently retrieved and reviewed again along with our inclusion criteria. As such, articles selected for data extraction were reviewed twice. The quality of the data was evaluated on a 2-point scale examining empirical or theoretical rigour and data relevance. A log within Covidence was recorded to track articles selected for exclusion.

### 3.5. Data Evaluation and Synthesis

As per Whittemore and Knafl [1], after the research identification and literature search have been performed, a comprehensive evaluation of the data must occur. While no specified method for evaluating the quality of data exists, the recommendation is that it should be customized to the type of studies included [1]. Journal Article Reporting Standards (JARS)-quantitative [21], JARS-qualitative and mixed-methods [22] criteria, and methods developed by Moher et al. [19] were used to analyze articles all articles included in this review.

During data analysis, studies were classified based on the type of evidence and analyzed sequentially for similarities and differences [1]. Similar findings were then compared and reduced into a succinct and manageable framework [1]. Articles identified through the quality assessment analysis as low relevance were excluded. After the data extraction process, two additional articles were removed due to a low-quality score not previously identified, and the remaining articles were kept for inclusion. Included studies were manually reduced and entered into a summary table (Table 2). An iterative process was undertaken to examine data, noting patterns, and themes, depicting relationships, and cluster similar variables to draw comparisons [1]. Patterns, commonalities, and differences were identified with frequent verification for accuracy to draw final conclusions [1].

## 4. Results

The final sample consisted of 18 articles published between 1992 and 2022, and designs including quantitative (*n* = 16 (88.9%)), integrative review (*n* = 1 (5.56%)), and a systematic review (*n* = 1 (5.56%)). Of the quantitative studies, most (*n* = 15 (83.3%)) were identified as cross-sectional. Some studies (*n* = 5 (27.8%)) did not identify their research design to be cross-sectional; however, due to the singular point of data collection at one point in time, they are cross-sectional in nature (e.g., [23, 24, 29, 31, 33]).

Among the included studies, themes were observed examining transformational leadership and intention to stay/intention to leave/turnover intention, and actual turnover as well as between transformational leadership and other variables including culture/climate of safety, organizational culture, organizational commitment, job satisfaction, and job stress.

### 4.1. Reported Scores

Three studies examined the disparities between staff nurses' perceptions of transformational leadership behaviors exhibited by nursing leaders and nurse leaders' own assessments of their transformational leadership behaviors. All three studies indicated that nursing leaders reported more frequent instances of transformational leadership than staff nurses did [28, 29, 32]. Among these, Goh et al. [28] established statistical significance for this difference (*ρ* < 0.05).

### 4.2. Culture, Climate, and Commitment

Laing et al. [30] established that transformational leadership positively influenced a safety-oriented climate, thereby enhancing the intention to remain. Ferreira et al. [27] corroborated these findings across four articles, showcasing how transformational leadership fosters a safety-driven culture or climate. Abualrub and Nasrallah [24] reported a robust positive correlation between transformational leadership behaviors and perceptions of organizational culture (*r* = 0.50, *ρ* ≤ 0.001), as well as the intention to remain in the workplace (*r* = 0.587, *ρ* ≤ 0.001). Brewer et al. [25] and Goh et al. [28] concurred, highlighting a favorable association between transformational leadership behaviors and organizational commitment (*r* = 0.495, *ρ* < 0.01; *rs* = 0.594, *ρ* < 0.05).

Several studies underline how transformational leadership cultivates a positive work climate and reinforces organizational commitment, consequently encouraging nurse's retention (e.g., [24–28, 30]). While Lyu et al. [31] found a moderate and significant relationship between organizational commitment and the intention to remain (*r* = 0.34, *ρ* ≤ 0.001), the data did not distinctly predict a staff nurse's intention to stay.

### 4.3. Job Satisfaction and Stress

Numerous studies reveal a positive correlation between transformational leadership behaviors and job satisfaction [6, 23, 28, 32]. Although Brewer et al. [25] noted that transformational leadership does not significantly forecast nurses' job satisfaction, Labrague et al. [6] and Lyu et al. [31] established a robust predictive link (*β* = 0.343, *ρ* ≤ 0.001; *β* = 0.562, *ρ* < 0.05). The positive nexus between job satisfaction and intention to stay is corroborated by multiple studies [22, 25, 31, 35]. Abualrub and Alghamdi [23] identified that transformational leadership accounted for 19% of the observed change in job satisfaction, while it explained only 2% of the variance in intent to stay [23].

In contrast to job satisfaction, additional research delved into job stress and burnout. Pishgooie et al. [16] unveiled a negative correlation between transformational leadership and job stress (*r* = −0.34, *ρ* < 0.001) and a positive connection between job stress and turnover intention (*r* = 0.34, *ρ* < 0.001). Theucksuban et al. [35] reported a negative association between burnout and intention to stay (*r* = −0.300, *ρ* < 0.01), with burnout elucidating 67.5% of the variation in nurses' intention to stay. While Ferreira et al. [27] could not discern a significant link between transformational leadership and burnout syndrome, their investigation into the separate facets of emotional exhaustion and cynicism revealed that transformational leadership tangibly and indirectly impacted these elements [27].

### 4.4. Intention to Stay

Research has consistently shown a connection between transformational leadership and the intent to stay in the workplace [24, 26, 31, 35, 36] or a converse link with turnover intention [7, 16, 37]. Cowden et al.'s [26] systematic review identified a relationship between transformational leadership and the intent to stay, although one of their included studies did not achieve statistical significance. Lavoie-Tremblay et al. [37] found a strong negative prediction of nurses' intention to leave healthcare facilities (*β* = −0.14, *ρ* < 0.05), yet no significant prediction of nurses' intent to leave the nursing profession. Lyu et al. [31] established transformational leadership as a substantial predictor of intent to stay (*β* = 0.793, *ρ* ≤ 0.001).

Furthermore, various studies underline that nurse managers' leadership styles account for 12% of the variance in anticipated nurse turnover, with transformational leadership significantly surpassing other studied styles [26, 34, 38]. However, Abualrub and Alghamdi [23] indicated that transformational leadership only accounted for 1% of the variation in intent to stay. Conversely, some studies suggest an insignificant relationship between transformational leadership and intent to stay [6, 23, 25, 30, 33].

Brewer et al. [25] found that transformational leadership indirectly influences intent to stay through organizational commitment and positive work environments. Goh et al.'s [28] study revealed that half of the surveyed hospital units showed a significant negative correlation between transformational leadership and turnover intention (ward A: *r* = −0.368, *ρ* < 0.01; ward D: *r* = 0.61, *ρ* < 0.01). Labrague et al. [6] echoed this, confirming a negative association between transformational leadership and organizational turnover intention (*r* = −0.080, *ρ* < 0.01). However, Kleinman [29] did not uncover a significant relationship between turnover intention and transformational leadership.

Transformational leadership also exerts an influence on intent to stay through the mediation of other variables. Laing et al. [30] established a positive impact of transformational leadership on safety climate, indirectly influencing intent to stay. Transformational leadership's positive indirect effect on intent to stay via the mediator of emotional intelligence was noted by Wang et al. [36] (*β* = 0.111, *ρ* ≤ 0.01). Lastly, beyond the intent to leave assessments, some studies analyzed actual turnover data. McDaniel and Wolf [32] found a turnover rate 5% lower than a magnet benchmark. Raup [33] observed actual nurse turnover to be 16% lower in units with nontransformational leadership.

## 5. Strengths and Limitations

### 5.1. Comprehensive Examination of Transformational Leadership and Retention

A significant number of studies have investigated the relationship between transformational leadership and nurse retention, often in conjunction with other variables. These studies have revealed varying frequencies of reported leadership behaviors, raising questions about the efficacy of displayed transformational leadership [29]. Nonetheless, despite these differences, Lavoie-Tremblay et al. [37] and Pishgooie et al. [16] underscore the importance of involving both nurses and nursing leadership in the assessment of transformational leadership behaviors. Such inclusive evaluation ensures a comprehensive understanding of the contextual dynamics at play, enhancing the accuracy of conclusions.

### 5.2. Cultivating Positive Organizational Culture

A positive culture within organizations can be gradually formed by leadership over time by systematically solidifying this culture through consistent transformational leadership behaviors [24]. A favorable culture empowers nurses by providing opportunities for decision-making, professional growth, and conflict resolution [24]. By actively seeking input, promoting engagement in decision-making, and embracing shared participation [26], nurse managers cultivate an atmosphere characterized by cooperation and collaboration, thus nurturing a culture of support [7]. This dynamic contributes to nurses' sense of commitment to their roles within the organization, ultimately bolstering retention.

### 5.3. Transformational Leadership and Job Satisfaction

The positive influence that transformational leadership behaviors has on nurses' career satisfaction is substantiated by several studies. As these transformational leadership behaviors look to support and coach nurses in their professional atmosphere, the transformational leader works to support a vision of nursing [32] and if this vision were to change, staff could report more dissatisfaction if they feel they need to change their vision to something they may not see as appropriate. Abualrub and Alghamdi [23] proposed that although job satisfaction may increase a nurse's intention to stay within nursing, it should be considered concurrently with other factors to maximize the potential to reduce actual turnover.

The interesting findings of Pishgooie et al. [16] regarding job stress warrant attention. Nurses reported lower levels of job stress than anticipated, suggesting a potential adaptive response to their demanding environment. However, the correlation between job stress and anticipated turnover emphasizes that even seemingly resilient individuals can be vulnerable to the negative effects of elevated stress levels. If the two major indicators of job stress (role clarity and conflict) become imbalanced, it can lead to the need to activate coping strategies, mitigate emotional exhaustion, and increase job dissatisfaction and anticipated turnover [16]. Given the strong correlation between anticipated turnover and actual turnover, the assessment of anticipated turnover emerges as a valuable tool for gauging the potential for future attrition. [16].

### 5.4. Impact of Transformational Leadership on Retention

While transformational leadership undoubtedly plays a pivotal role in retention [29], it may not have a large enough impact on a nurse's decision to leave the profession. Transformational leadership involves creating opportunities and adapting to organizational change to meet demanding needs [7]. The elements of transformational leadership are important to use for all nurses and are especially important to use as a framework to address the needs of novice nurses [7, 37]. The structured support and mentorship embedded in transformational leadership practices are particularly beneficial for novice nurses, facilitating their professional development. Despite its undeniable relevance, it is important to acknowledge that transformational leadership, while impactful, may need to be complemented by other strategies to achieve significant reductions in both anticipated turnover and intention to quit. These measures are vital for mitigating potential consequences such as decreased productivity and increased organizational costs [27].

In summary, the extensive body of research underlines the central role of transformational leadership behaviors in nurse retention. However, this influence is intertwined with a complex web of organizational, cultural, and individual factors. By embracing transformational leadership as a core principle while addressing these multifaceted dynamics, healthcare institutions can optimize nurse retention, ensuring a positive impact on both the organization and the nursing profession as a whole.

## 6. Implications for Nursing Management

To foster positive outcomes within the nursing organization, effective leadership skills are pivotal for both nursing staff and leadership members. These skills encompass proficient communication, the ability to influence, inspire, and motivate others, and the facilitation of decision-making opportunities. Multiple studies emphasize the significance of robust nursing leadership training to cultivate transformational leadership behaviors that enhance nursing retention [6, 16, 24, 26, 27, 34, 36, 37].For instance, Theucksuban et al. [35] highlighted the necessity for comprehensive training programs, encompassing areas such as transformational leadership methods, human resource training, and strategic planning, which contribute to the observed elevation in reported transformational leadership behaviors. However, it is imperative to align these behavioral changes with the organizational culture across all tiers [26], underscoring the pivotal role of leadership development programs in promoting favorable leadership behaviors and making sure that they fit well with the organization's values and beliefs.

## 7. Conclusion

In conclusion, the examined literature consistently revealed that nursing leaders often reported higher frequencies of transformational leadership behaviors than staff nurses. Positive links between transformational leadership and culture/climate, organizational commitment, and job satisfaction were evident, while job stress and burnout showed varying correlations. Transformational leadership exhibited connections with intent to stay, although this influence was tempered by other variables. While transformational leadership plays a pivotal role in fostering retention, its impact may need to be complemented by other strategies. Further research is needed to comprehensively assess the impact of transformational leadership on nurse retention, thereby enhancing our understanding and promoting positive workplace outcomes. The implementation of interventional studies, encompassing pre- and postevaluations of organizational outcomes, creates valuable insights for the design and refinement of nursing leadership training programs.

The implications for nursing management are clear: leadership development programs are instrumental in promoting favorable leadership behaviors, but their alignment with the organizational ethos is crucial. Encouraging leadership training tailored to desired outcomes, fostering self-awareness among nurse leaders, and nurturing open communication channels are recommended strategies. By leveraging these insights, nursing leaders can not only enhance their own transformational leadership skills but also foster a positive workplace culture that contributes to nurse retention and, ultimately, the success of the organization.

## 8. Recommendations

The implementation of self-awareness measures within nursing leadership could address the reported disparities in scores, given that self-awareness is a cornerstone of effective leadership [28]. Proactive strategies to mitigate burnout syndrome should be created, including initiatives that acknowledge and appreciate employees' strengths [27]. The establishment of two-way communication models between nurses and nurse leaders, facilitating an open avenue for expressing concerns, adversities, and organizational issues, is recommended [35]. Organizations are encouraged to tailor leadership training programs to their desired outcomes, recognizing the positive impact of transformational leadership on patient, professional, and institutional levels [27].

The insights gained from this integrative review clearly demonstrate that nursing leaders are well-positioned to not only strengthen their own transformational leadership capabilities, but also institute measures that inspire a positive workplace culture and elevate nursing staff experiences.

## Figures and Tables

**Table 1:** Search results (June 8, 2022).

Search equations (in sequential order of completed searches)	Filters	Identified records	Number of duplicate records
PubMed	((Transformational leadership [Title/Abstract]) AND (nurs^*∗*^ [Title/Abstract] OR RN [Title/Abstract])) AND (attrition [Title/Abstract] OR retention [Title/Abstract] OR turnover [Title/Abstract] OR “intent to stay” [Title/Abstract] OR “intent to leave” [Title/Abstract] OR “intent to quit” [Title/Abstract] OR “turnover intention” [Title/Abstract])	54	1
MEDLINE via Ovid	“Transformational leadership”.ti, ab, kw. (nurs^*∗*^ or RN).ti,ab,kw. (retention or attrition or turnover or “intent to stay” or “intent to leave” or “intent to quit” or “turnover intention”). ti, ab, kw	52	52
ProQuest	AB, TI, IF (“transformational leadership”) AND AB, TI, IF (nurs^*∗*^ OR RN) AND AB, TI, IF (retention OR attrition OR turnover OR “intent to stay” OR “intent to leave” OR “intent to quit” OR “turnover intention”)	98	32
CINAHL	[TI “transformational leadership” OR AB “transformational leadership”] AND [TI (nurs^*∗*^ or RN) OR AB (nurs^*∗*^ or RN)] AND [TI (retention or attrition or turnover or “intent to leave” or “intent to stay” or “intent to quit” or “turnover intention”) OR AB (retention or attrition or turnover or “intent to leave” or “intent to stay” or “intent to quit” or “turnover intention”)]	93	57

**Table 2:** Literature summary.

Author/year/country	Study aim	Study design	Methods of measurement	Statistical analyses	Sample	Conclusions/implications
Abualrub and Alghamdi [23] Saudi Arabia	To examine the impact of leadership styles of nurse managers on Saudi nurses' job satisfaction and their intent to stay at work	Descriptive, cross-sectional/correlational	Demographic form The multifactor leadership questionnaire (MLQ-5X) Job satisfaction survey (JSS) The McCain's intent-to-stay scale (MISS) Collected via paper	Descriptive Pearson correlation Hierarchical regression Significance 0.05	Convenience sampling *n* = 308 (51.3% response rate) RNs from 6 public hospitals in Western Region of Saudi Arabia with at least 6 months of experience at their current job and under the direct supervision of a nurse manager	Nurse administrations should promote the importance of a transformational leadership style in increasing job satisfaction and thereby increasing intention to stay among nurses
Abualrub and Nasrallah [24] Jordan	To investigate the impact of leadership behaviors of nurse managers and organizational culture on Jordanian nurses' intention to stay at work in public, private, and university hospitals	Descriptive, correlational, cross-sectional, comparative	A sociodemographic form The leadership practice inventory The professional organizational culture (POC) scale The McCain's intent-to-stay scale (MISS) Methods of data collection unclear	Descriptive Pearson product-moment ANOVA Hierarchical multiple regression Significance 0.05 Data cleaned/screened and treated outliers	Convenience sampling *n* = 285 (81% response rate) Nurses from 6 hospitals (public, private, and university-affiliated) in Jordan	The study recommends that organizations should focus on the promotion of transformational leadership through nursing education programs and continuous education programs that also focus on organizational culture as these two variables together can have a large impact on the intention to stay
Brewer et al. [25] United States of America	To examine the effect of transformational leadership on early career nurses' intent to stay, job satisfaction, and organizational commitment	Cross-sectional correlational (with descriptive data)	Demographic survey An expanded version of the Price model of turnover was used	Descriptive Ordered probit model	No stated sampling technique *n* = 1037 (69% response rate) RNs licensed for 7.5–8.5 years who worked in the healthcare field and collaborated with MD's	Transformational leadership has the potential to decrease attrition through a positive work environment and increased organizational commitment. Workplaces should also invest in systems change that promotes these
Cowden et al. [26] Canada	To describe the findings of the literature that examines the relationship between managers' leadership practices and staff nurses' intent to stay in their current position	Systematic review	A systematic review of six electronic databases followed by manual searches through specific nursing journals. English articles published between 1985–2010 examining manager leadership and staff nurse's intent to stay were included in the review	Manuscripts reviewed twice against inclusion criteria for relationships and quality. Used adapted tools for quality assessment and critical appraisal. Two reviewers	A total of 23 studies were included in the review and were rated as moderate or strong in quality. Twenty-two of the studies included were quantitative and 1 study was qualitative	Nurse managers who practice relational leadership (e.g., transformational leadership) and ensure positive work environments foster nurse's intention to stay. This review provides a solid foundation for advancing current conceptual models that incorporate leadership theory into management practices
Ferreira et al. [27] Brazil	To map leadership styles that positively impact patients, professionals, and institutions	Integrative review	An integrative review of selected articles was chosen from five databases. Two researchers assessed the quality following the Joanna Briggs Institute's (JBI) recommendations	Four researchers performed analysis using the Preferred Reporting Items for Systematic Reviews and Meta-Analysis. Quality assessed by two researchers using Joanna Briggs Institute's recommendations. Information was deduced into a literature summary table	A total of 35 articles were included in the review. 32 quantitative articles, 2 qualitative articles, and 1 quasiexperimental article were included in the review	The results show the need for nurses to improve their leadership skills through training and development programs in transformational leadership to achieve positive results. Transformational leadership was shown to reduce absenteeism at work and decrease the intention of professionals to quit their jobs
Goh et al. [28] Singapore	To assess the leadership styles of nurse leaders, as perceived by their employees, to explore differences between self- ratings and others' ratings of leadership styles, and to determine if there is a correlation between perceived leadership styles and organizational outcomes	Cross-sectional/with comparative, correlational, and descriptive data	Demographic questionnaire The multifactor leadership questionnaire (MLQ-5X) The organizational commitment The three-index item questionnaire	Descriptive Spearman's rho One-sample *t*-test Significance 0.05 Reported reliability and validity	Convenience sample *n* = 111 (37% response rate) RNs from 4 inpatient wards at an acute tertiary care hospital	Overall, registered nurses reported that their nurse leaders predominately exhibited both transformational and transactional leadership. Nurse leaders in this study tend to rate themselves higher than others rate them. The results identify the need for self-awareness elements in nursing leadership development programs. Transformational leadership has the ability to increase organizational commitment and decrease turnover intention
Kleinman [29] United States of America	To describe perceptions of managerial leadership behaviors associated with staff nurse turnover and to compare nurse manager leadership behaviors as perceived by managers and their staff nurses	Stated it was a prospective, correlational design and then also stated it was a descriptive, correlational design. Inferenced to be cross-sectional	Demographic questionnaire The multifactor leadership questionnaire (MLQ-5X) Nurse turnover was measured as the percentage of staff nurses who had resigned from the medical center during the 6-month period from January through June 2003	Descriptive Pearson product-moment correlation ANOVA Calculated Cronbach's alpha Significance 0.05	Sampling technique not stated *n* = 79 staff nurses and 10 nurse managers (response rate 25% for staff nurses and 62% for nurse managers) Conducted at a 465-bed community hospital	Transformational leadership was not correlated with staff turnover. However, nurse managers perceived they had higher frequencies of transformational leadership behaviors than staff nurses. Therefore, it is important for nursing managers to be visible to staff nurses which includes schedule adjustments to allow managers to interact with nurses who do not work the day shift
Labrague et al. [6] Philippines	Examined the influence of toxic and transformational leadership practices on nurses' job satisfaction, psychological distress, absenteeism, and intent to leave the organization or the nursing profession	Cross-sectional (descriptive and correlational statistics)	Did not state they handed out demographic surveys; however, they created descriptive statistics based on the demographic information The toxic leadership behaviors of nurse managers scale The global transformational leadership (GTL) scale Job satisfaction index Intention to quit scale adapted (two single-item measures) developed by O'Driscoll and Beeher (1994) Researcher-designed single-item question for absenteeism	Descriptive Pearson correlation Hierarchical multiple regression reported assumptions testing Reported reliability and validity	Sampling technique not stated *n* = 770 (response rate 86%) RN's employed in 15 hospitals with at least 6 months of experience	Nurses perceived their nurse managers are highly transformational. This study indicated transformational leadership to have a positive effect on job satisfaction and turnover intention. Therefore, nurse retention strategies should include measures to increase transformational leadership and decrease toxic leadership in nurse managers through evidence-based education, training, professional development opportunities, and utilizing leadership assessment tools when considering nurse manager candidates

Laing et al. [30] Taiwan	To propose a theoretical model and apply it to examine the structural relationships among nurse characteristics, leadership characteristics, safety climate, emotional labour, and intention to stay for hospital nurses	Cross-sectional	Demographic survey The multifactor leadership questionnaire (MLQ-5X) The safety attitudes questionnaire (SAQ) The emotional labour questionnaire The intention to stay scale (ITS)	Descriptive Structural equation modelling Confirmatory factor analysis model Significance set a priori for all statistical calculations. Reported reliability and calculated validity	Purposive sample *n* = 414 (91.6% response rate) Purposely selected 2 regional hospitals in Yilan county (1 public and 1 private)	Intention to stay positively affected safety climate and transformational was found to have an indirect effect on the intention to stay. This indirect effect was also mediated separately by emotional labour and safety climate. The study recommends that administrators encourage nurse managers to adopt transformational leadership to strengthen perceptions of a positive safe climate in the workplace, resulting in increased intention to stay
Lavoie-Tremblay et al. (2015) Canada	To investigate the impact of nurse managers exercising transformational vs. abusive leadership practices with novice nurses	Predictive, cross-sectional	Did not state they handed out demographic surveys; however, they created descriptive statistics based on the demographic information. The global transformational leadership (GTL) scale Abusive leadership scale Intention to quit scale adapted (two single-item measures) developed by O'Driscoll and Beeher (1994) Quality of care scale	Descriptive Linear regression Confirmatory factor analysis model Maximum likelihood Reported reliability and validity	Random sampling *n* = 727 (20.8% response rate) Novice RN's (less than 5 years' experience) and the ability to read French	Transformational leadership strongly predicted quality of care scores and intention to quit healthcare facilities. whereas abusive leadership predicts a nurse's intention to quit the profession. Implications should include the promotion and training of transformational leadership and the reduction of abusive leadership to assist with the nursing shortage which can also increase the quality of patient care
Lyu et al. [31] China	To examine the level of intention to stay and the influence of ten predictors (transformational leadership, career growth, workgroup cohesion, educational level, monthly income, professional position, years of experience, gender role conflict, organizational commitment, and job satisfaction) on intention to stay	Descriptive predictive-cross-sectional	Demographic data profile The McCain's intent-to-stay scale (MISS) The leadership practice inventory The career growth of nurse scale The group cohesion scale (GCS) The gender role conflict short-form The three-component model (TCM) employee commitment survey The McCloskey/Mueller satisfaction scale	Descriptive Chi-square Biserial correlation coefficient Significance 0.05 Reported assumptions testing Reported reliability	Sampling technique not stated *n* = 430 (response rate 89.6%) Male nurses working in five university hospitals for at least one year	Work-group cohesion, career growth, transformational leadership, and job satisfaction significantly predicted a nurse's intention to stay in their position. This suggests nursing administrations and policymakers to develop interventions (e.g., training programs) to improve these four modifiable factors
Magbity et al. [7] Ghana	Investigated the leadership styles of nurse managers' impact on turnover intention among nurses in hospitals	Descriptive, cross-sectional/correlational	Demographic data were not collected The multifactor leadership questionnaire (MLQ-5X) The turnover intention scale (TIS-6)	Descriptive Pearson product-moment Multiple regression analysis	Sampling technique not noted *n* = 250 (response rate not noted) Nurses employed within 5 distinctive hospitals (assumed to be in Ghana but not specified)	Transformational and participative leadership styles decrease turnover intention whereas autocratic and laissez-faire increase turnover intention. Nurse managers and administrators should emphasize a positive workplace climate and evidence-based leadership practices such as transformational and participative leadership to reduce staff turnover
McDaniel and Wolf [32] United States of America	To determine whether transformational theory applies to nurses in an entire nursing service department	Descriptive, comparative, cross-sectional	Demographic data not collected The multifactor leadership questionnaire (MLQ-5X) The work satisfaction scale Turnover data were obtained from the nurse service reported monthly Collected via mail	Descriptive t-tests Pearson correlation Reported validity and reliability Significance 0.05	Sampling technique not noted *n* = 46 registered nurses, 9 midlevel administrators, and 1 nurse executive (60%, 81%, and 100% response rates) Conducted on one unit at a moderate-sized facility	Transformational leadership was positively correlated with job satisfaction as well as the turnover data for registered staff is lower in environments where leaders have predominately transformational leadership behaviors. Nurse executives should recruit employees demonstrating transformational characteristics and create structures that will support and facilitate these behaviors
Pishgooie et al. [16] Iran	To investigate the relationship between leadership style and nurse job stress and anticipated turnover	Cross-sectional/correlational	Demographic questionnaire The multifactor leadership questionnaire (MLQ-5X) The health and safety executive questionnaire The anticipated turnover scale (ATS)	Descriptive Pearson correlation Reported assumptions testing Reported reliability Significance 0.05	Randomised multistage sampling and simple random sampling *n* = 1617 (96.76% response rate) All nurses working in 10 government (otherwise known as public) hospitals were randomly selected based on geographical location and size. RN's with 1 year of experience not in a management position	Nurse leaders can improve transformational leadership behaviors and reduce turnover by developing training programs and creating a supportive work environment. Nurse leaders should also provide a supportive work environment to support intention to stay
Raup [33] United States of America	To examine the impact of leadership styles used by emergency department (ED) nurse managers in academic health centers on nurse turnover and patient satisfaction as measured by the full-range leadership model	Descriptive, cross-sectional	Demographics survey The multifactor leadership questionnaire (MLQ-5X) Staff turnover rates were self-reported by ED nurse managers using statistics provided by the nurse managers	Descriptive Fisher's exact tests	Convenience sampling *n* = 45 (15.3% response rate) Attempts were made to contact the ED managers of the primary hospitals identified by each of the 101 academic health centers over a 20-month study period. 15/98 possible sites returned questionnaires	This study did not find a statistically significant difference in leadership style on staff nurse retention. However, the transformational leadership style may offer some benefits to both managers and staff nurses in high-stress areas such as the emergency department including increased retention
Suliman et al. [34] Jordan	To assess the effect of nurse managers' leadership styles on predicted nurse turnover	Descriptive, cross-sectional/correlational	Demographic questionnaire The multifactor leadership questionnaire (MLQ-5X) The anticipated turnover scale (ATS)	Descriptive ANOVA Multiple regressions One-sample *t*-test Significance 0.05	Convenience sampling *n* = 250 (89% response rate) Nurses employed in three public sector hospitals and one university-affiliated (teaching hospital)	Transformational leadership has a significant role in decreasing nurses' anticipated turnover, therefore training programs, nursing management courses at undergraduate and postgraduate levels, and clinical training of nursing students should focus on this
Theucksuban et al. [35] Thailand	To test the causal model of intent to stay in employment of nurses in regional medical centers	Cross-sectional (with correlational and descriptive statistics)	Demographic data form The McCain's intent-to-stay scale (MISS) The leadership practice inventory The social integration scale The nursing activity scale The promotional opportunity scale The Maslach burnout inventory The job satisfaction scale The organizational commitment questionnaire	Descriptive Structural equation modelling Maximum likelihood Reported assumptions testing Reported reliability Significance 0.01	Multistage random sampling *n* = 1224 (95.84% response rate) RN's with more than one year of experience, providing direct care to the obstetrics, surgical, medical, pediatric, operating room, intensive care, or emergency departments in nine regional medical centers	Transformational leadership, coworker support, professional autonomy, opportunities for promotion, marital status, and job satisfaction positively affected intent to stay, whereas burnout negatively affected intent to stay. These seven factors that should be considered by nurse managers in developing a framework for creating interventions to promote intention to stay. Policymakers should consider enacting policies and regulations for nurses' benefits to increase nurses' intent to stay
Wang et al. [36] China	To examine the role of staff nurse emotional intelligence between transformational leadership and nurse intent to stay	Cross-sectional descriptive design	Demographic survey Wong and Law emotional intelligence scale (WLEIS) The transformational leadership scale (Chinese version) The nurse intention to stay scale by Tao and Wang	Descriptive Structural equation modelling Maximum likelihood Significance 0.05	Convenience sampling *n* = 535 (85.9% response rate) RNs from four general hospitals working for at least 1-year full time at the study hospitals	Transformational leadership directly and indirectly affects intention to stay as well as emotional intelligence. Nurse leaders and policymakers should develop training programs for nurse managers, school education, and continuing education programs to foster transformational leadership and nurse's emotional intelligence to aid in retention

## Data Availability

The data that support the findings of this study are included in the supplementary material of this article.

## Supplementary Materials

Supplementary Materials Supplementary materials are presented for the reporting of the PRISMA checklist.

## References

[1] Whittemore R., Knafl K. (2005). The integrative review: updated methodology. *Journal of Advanced Nursing*.

[2] Cardiff S., Gershuni O., Giesbergen-Brekelmans A. (2023). How local, first-line nurse leaders can positively influence nurse intent to stay and retention: a realist review. *Journal of Clinical Nursing*.

[3] McCay R., Lyles A., Larkey L. (2018). Nurse leadership style, nurse satisfaction, and patient satisfaction. *Journal of Nursing Care Quality*.

[4] Ystaas L. M. K., Nikitara M., Ghobrial S., Latzourakis E., Polychronis G., Constantinou C. S. (2023). The impact of transformational leadership in the nursing work environment and patients’ outcomes: a systematic review. *Nursing Reports*.

[5] Mckenna J., Jeske D. (2020). Ethical leadership and decision authority effects on nurses’ engagement, exhaustion, and turnover intention. *Journal of Advanced Nursing*.

[6] Labrague L. J., Nwafor C. E., Tsaras K. (2020). Influence of toxic and transformational leadership practices on nurses’ job satisfaction, job stress, absenteeism and turnover intention: a cross-sectional study. *Journal of Nursing Management*.

[7] Magbity J. B., Ofei A. M. A., Wilson D. (2020). Leadership styles of nurse managers and turnover intention. *Hospital Topics*.

[8] Al-Thawabiya A., Singh K., Al-Lenjawi B. A., Alomari A. (2023). Leadership styles and transformational leadership skills among nurse leaders in Qatar, a cross-sectional study. *Nursing Open*.

[9] Hult M., Teramo-Moisio A., Kaakinen P. (2023). Relationships between nursing leadership and organizational, staff and patient outcomes: a systematic review of reviews. *Nursing Open*.

[10] Avolio B. J., Bass B. M. (2004). *Multifactor Leadership Questionnaire: Manual and Sample Set*.

[11] Marufu T. C., Collina A., Vargas L., Gillespie L., Almghairbi D. (2021). Factors influencing retention among hospital nurses: systematic review. *British Journal of Nursing*.

[12] Bass B. (1995). Theory of transformational leadership redux. *The Leadership Quarterly*.

[13] Aydogdu (2023). Trends of publications on transformational leadership in nursing: a bibliometrics analysis. *Leadership in Health Services*.

[14] Lappalainen M., Härkänen M., Kvist T. (2020). The relationship between nurse manager’s transformational leadership style and medication safety. *Scandinavian Journal of Caring Sciences*.

[15] Gebreheat G., Teame H., Costa E. I. (2023). The impact of transformational leadership style on nurses’ job satisfaction: an integrative review. *SAGE Open Nursing*.

[16] Pishgooie A. H., Atashzadeh-Shoorideh F., Falcó-Pegueroles A., Lotfi Z. (2019). Correlation between nursing managers’ leadership styles and nurses’ job stress and anticipated turnover. *Journal of Nursing Management*.

[17] Robbins B., Davidhizar R. (2020). Transformational leadership in health care today. *The Health Care Manager*.

[18] Richardson W. S., Wilson M. C., Nishikawa J., Hayward R. S. (1995). The well-built clinical question: a key to evidence-based decisions. *ACP Journal Club*.

[19] Moher D., Liberati A., Tetzlaff J., Altman D. G. (2009). Preferred reporting items for systematic reviews and meta-analyses: the PRISMA statement. *British Medical Journal*.

[20] Kellermeyer L., Harnke B., Knight S. (2018). Covidence and rayyan. *Journal of the Medical Library Association*.

[21] Applebaum M., Kline R. B., Nezu A. M., Cooper H., Mayo-Wilson E., Rao S. M. (2018). Journal article reporting standards for quantitative research in psychology: the APA publications and communications board task force report. *American Psychologist*.

[22] Levitt H. M., Bamberg M., Creswell J. W., Frost D. M., Josselson R., Suárez-Orozco C. (2018). Journal article reporting standards for qualitative primary, qualitative meta-analytic, and mixed methods research in psychology: the APA publications and communications board task force report. *American Psychologist*.

[23] Abualrub R., Alghamdi M. G. (2012). The impact of leadership styles on nurses’ satisfaction and intention to stay among Saudi nurses. *Journal of Nursing Management*.

[24] Abualrub R. F., Nasrallah M. A. (2017). Leadership behaviours, organizational culture and intention to stay amongst Jordanian nurses. *International Nursing Review*.

[25] Brewer C. S., Kovner C. T., Djukic M. (2016). Impact of transformational leadership on nurse work outcomes. *Journal of Advanced Nursing*.

[26] Cowden T., Cummings G., Profetto-McGrath J. (2011). Leadership practices and staff nurses’ intent to stay: a systematic review: leadership practices and staff nurses’ intent to stay. *Journal of Nursing Management*.

[27] Ferreira T. D. M., Reis de Mesquita G., Cipriane de Melo G. (2022). The influence of nursing leadership styles on the outcomes of patients, professionals and institutions: an integrative review. *Journal of Nursing Management*.

[28] Goh A. M. J., Ang S. Y., Della P. R. (2018). Leadership style of nurse managers as perceived by registered nurses: a cross-sectional survey. *Proceedings of Singapore Healthcare*.

[29] Kleinman C. (2004). The relationship between managerial leadership behaviors and staff nurse retention. *Hospital Topics*.

[30] Laing H., Tang F., Wang T., Lin K., Yu S. (2016). Nurse characteristics, leadership, safety climate, emotional labour and intention to stay for nurses: a structural equation modelling approach. *Journal of Advanced Nursing*.

[31] Lyu X., Akkadechanunt T., Soivong P., Juntasopeepun P. (2022). Factors influencing intention to stay of male nurses: a descriptive predictive study. *Nursing and Health Sciences*.

[32] McDaniel C., Wolf G. A. (1992). Transformational leadership in nursing service: a test of theory. *The Journal of Nursing Administration: The Journal of Nursing Administration*.

[33] Raup G. H. (2008). The impact of ED nurse manager leadership style on staff nurse turnover and patient satisfaction in academic health center hospitals. *Journal of Emergency Nursing*.

[34] Suliman M., Almansi S., Mrayyan M., AlBashtawy M., Aljezawi M. (2020). Effect of nurse managers’ leadership styles on predicted nurse turnover. *Nursing Management*.

[35] Theucksuban B., Wichaikhum O., Kunaviktikul W., Abhicharttibutra K. (2011). Testing a model of Thai nurses’ intent to stay in employment. *International Nursing Review*.

[36] Wang L., Hong T., Bowers B. J., Brown R., Zhang Y. (2018). When nurse emotional intelligence matters: how transformational leadership influences intent to stay. *Journal of Nursing Management*.

[37] Lavoie-Tremblay M., Fernet C., Lavigne G. L., Austin S. (2015). Transformational and abusive leadership practices: impacts on novice nurses, quality of care and intention to leave. *Journal of Advanced Nursing*.

[38] Boyle D. K., Bott M. J., Hansen H. E., Woods C. Q., Taunton R. L. (1999). Managers’ leadership and critical care nurses’ in- tent to stay. *American Journal of Critical Care*.

